# Complete Genome Sequence of *Ehrlichia muris* Strain AS145^T^, a Model Monocytotropic *Ehrlichia* Strain

**DOI:** 10.1128/genomeA.01234-13

**Published:** 2014-01-30

**Authors:** Nagaraja R. Thirumalapura, Xiang Qin, Jeeba A. Kuriakose, David H. Walker

**Affiliations:** a Department of Pathology, University of Texas Medical Branch, Galveston, Texas, USA; b Center for Biodefense and Emerging Infectious Diseases, University of Texas Medical Branch, Galveston, Texas, USA; c Sealy Center for Vaccine Development, University of Texas Medical Branch, Galveston, Texas, USA; d Institute for Human Infections and Immunity, University of Texas Medical Branch, Galveston, Texas, USA; e Human Genome Sequencing Center, Baylor College of Medicine, Houston, Texas, USA

## Abstract

We report here the complete genome sequence of *Ehrlichia muris* strain AS145^T^, which was isolated from a wild mouse in 1983 in Japan. *E. muris* establishes persistent infections in laboratory mice and is widely used as a surrogate pathogen in a murine model of ehrlichiosis.

## GENOME ANNOUNCEMENT

*Ehrlichia* are tick-borne Gram-negative bacterial pathogens that belong to the family *Anaplasmataceae* within the order *Rickettsiales* and the class *Alphaproteobacteria*. Human ehrlichial pathogens include *Ehrlichia chaffeensis*, *Ehrlichia ewingii*, and a recently discovered new species, *Ehrlichia muris*-like agent (EMLA) (1–3). *Ehrlichia canis* and *Ehrlichia ruminantium* cause disease in canines and ruminants, respectively (4). *E. chaffeensis* and *E. ewingii* also cause disease in dogs. *E. chaffeensis* and *E. ewingii* do not cause disease in immunocompetent mice; thus, surrogate murine pathogens *E. muris* and *Ixodes ovatus* ehrlichia (IOE) are used in animal models of ehrlichiosis (5, 6). The complete genome sequences of *E. chaffeensis* strain Arkansas, *E. canis* strain Jake, and three strains of *E. ruminantium* were previously reported (7–9). Here, we report the complete genome sequence of *E. muris* strain AS145^T^.

*E. muris* AS145^T^ (strain Asuke) was a gift from Yasuko Rikihisa, Ohio State University, Columbus, OH. *E. muris* was first isolated from the spleen of a wild mouse (*Eothenomys kageus*) in in 1983 in Japan (10, 11). The strain has been maintained in the laboratory by continuous passage in a DH82 canine monocytic cell line. For sequencing of the genome, we reisolated *E. muris* from the spleens of infected C57BL/6 mice and cultured in DH82 cells. The bacteria were purified by Percoll density-gradient centrifugation, and genomic DNA was extracted using the DNeasy kit (Qiagen, Valencia, CA). The genome was then sequenced using the Roche GS FLX system (Eurofins MGW Operon, Huntsville, AL). An 8-kb-long paired-end library was prepared from the genomic DNA, and a total of 273,255 reads were generated. Assembly of the reads using the MIRA Assembler version 3.2.1 resulted in 15 scaffolds containing 35 contigs and 1,204,716 bases. Twelve large contigs with an average size of about 98,216 bases contain 97% of the bases. The gaps in the genome were closed by PCR amplification of the regions, followed by sequencing of the amplicons and integration into the assembly. A single contig status was achieved for the genome, and the circular chromosomal structure was validated.

The genome of *E. muris* consists of a single circular chromosome containing 1,196,717 bp, with 30% G+C content. The origin of replication (*oriC*) was predicted to a 423-nucleotide region upstream of a hypothetical protein (locus tag EMUR_0005) using the Ori-Finder program, and 1 bp was set as the beginning of the *oriC* (12). The genome was annotated by the NCBI Prokaryotic Genome Annotation Pipeline version 2.0, which uses GeneMarkS+ for gene prediction (http://www.ncbi.nlm.nih.gov/genome/annotation_prok/). The genome of *E. muris* is predicted to contain 964 genes, including 904 protein-coding sequences (CDSs), one copy of the each of the rRNA genes (5S, 16S, and 23S), 37 tRNA genes, 19 pseudogenes, one small noncoding RNA, and 16 frameshifted genes. The availability of the complete genome of *E. muris* will allow comparative analysis with other ehrlichial genomes, particularly with the closely related *E. muris*-like agent, an emerging human pathogen.

### Nucleotide sequence accession number.

The *E. muris* strain AST145^T^ genome sequence has been deposited in GenBank under the accession no. CP006917.
